# Does Use of Solid Cooking Fuels Increase Family Medical Expenses in China?

**DOI:** 10.3390/ijerph19031649

**Published:** 2022-01-31

**Authors:** Boqiang Lin, Kai Wei

**Affiliations:** School of Management, China Institute for Studies in Energy Policy, Collaborative Innovation Center for Energy Economics and Energy Policy, Xiamen University, Xiamen 361005, China; kaiwei020@gmail.com

**Keywords:** solid fuels, fuels transition, medical expense, residential health

## Abstract

China has tried to replace solid fuels with cleaner energy in households. The benefits of fuel switching need to be identified. This article shows that households using solid cooking fuels suffer heavier medical expenses than those using non-solid cooking fuels. After accounting for family characteristics, using solid fuels is associated with 1.4–1.9% increases in medical care. Through the analysis of the impact mechanism, we found that solid cooking fuels harm the health conditions of family members and increase the probability of illness, thereby increasing medical expenses, while the ratio of fuel fees does not change significantly if switching cooking fuels. Furthermore, we explored heterogeneity to better understand the underlying relationship. For urban and higher-educated families with house ownership, the impact of solid fuels on medical expenses was weaker compared to rural and lower-educated households without owned houses. Therefore, considering the costs and benefits, we recommend continuing the conversion from solid fuels to non-solid fuels. In the fuel transition process, it is beneficial to raise residents’ awareness and improve behavior to avoid indoor air pollution.

## 1. Introduction

Countries around the world have made considerable improvements in household energy supply during the past decades. However, there are still more than 2 billion people who do not have access to clean cooking facilities [1]. Every year, about 2.5 to 3 million people die prematurely due to indoor pollution, and the use of solid fuels is the main reason of household air pollution [2]. Especially in developing countries, the problems caused by solid fuels have negative influences on the well-being of humans. On the one hand, many people spend significant amounts of time collecting firewood, leaving little time for exercise or recreation. On the other hand, the use of firewood harms the health status of household residents, especially women and children, who spend most of their time indoors.

**Figure 1:**
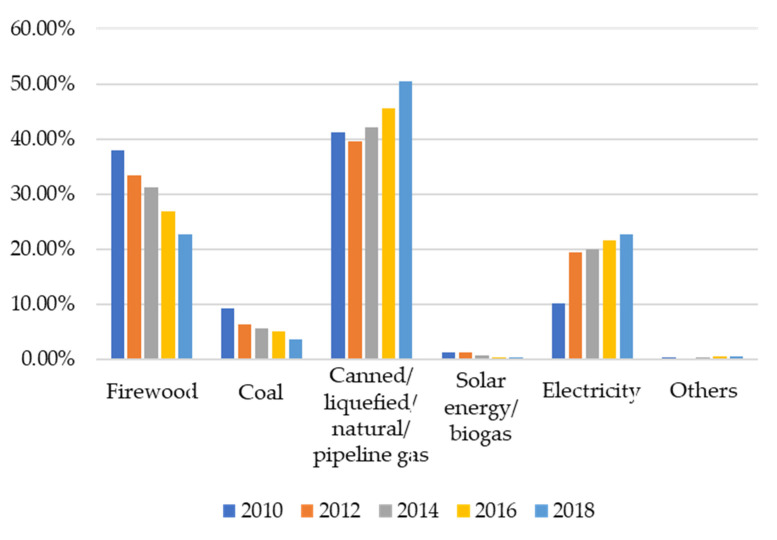
Changes in the proportion of household main cooking fuels from 2010 to 2018.

The International Energy Agency (IEA) [1] recently reported that the number of people without access to electricity all over the world had steadily declined from 1680 million in 2000 to 770 million in 2019. China also has made a significant breakthrough in the accessibility of electricity services. In 2015, China achieved full coverage of electricity, and the per capita electricity consumption has increased year by year [3,4,5]. However, many households still cannot use clean cooking fuel and equipment for cooking activities [6]. According to China’s Family Panel Studies (CFPS), the household main cooking fuel source had undergone considerable changes from 2010 to 2018. As shown in Figure 1, the use ratio of solid cooking fuels, including firewood and coal, decreased from 47.15% to 26.2%, while the use ratio of clean non-solid cooking fuels, including canned gas, liquefied gas, natural gas, pipeline gas, electricity, solar energy and biogas increased from 52.46% to 73.29%. According to the World Health Organization’s (WHO) Household Energy database, population access to clean fuels and technologies for cooking accounted for 59.26% of the total population in 2016. Even though the proportion of solid fuels dropped significantly, more than a quarter of households still used solid fuels in 2018.

As a typical poverty phenomenon, energy poverty has always been a hot issue in the field of energy. The definition of energy poverty generally has two aspects: including the accessibility to clean fuels and the affordability of fuel fees [7,8]. For developing countries, energy poverty indicates a lack of electricity, clean fuels and energy facilities, that is, a high dependence on traditional solid fuel [9]. High fuel costs as well as the inability to use modern fuels are both considered signs of energy poverty [10,11].

Moreover, studies have shown that the use of solid fuels by the residential sector impacts economic development, environmental quality, physical and mental health. People are already aware of the importance of addressing climate change and are making the energy transition in various sectors [12]. The use of clean fuels in the residential sector can reduce greenhouse gas emissions [13,14] and fuel replacement programs in the residential sector are attracting attention. Therefore, it is of great significance to discuss the impact of solid fuel household use and explain reasons for promoting clean fuels. Moreover, the benefits and costs during the evolution towards a low-carbon society should be considered [8].

The main contributions of this article are three-fold. First, based on national micro-survey data in China, we use econometric empirical methods to analyze the impact of solid fuel use on residents’ lives from the perspective of household medical expenses. Previous studies mainly focused on the measurement of energy poverty or solid fuel use [15,16,17], or the impact of energy poverty on residents’ physical and mental health [17,18]. Some studies examined the effects of environmental air pollution, treatment-seeking behavior and health insurance on medical cost [19,20,21], but studies seldom paid attention to the effect of solid fuel use on medical expenses. Some residential households refuse to abandon traditional solid cooking fuels, and are concerned about the increased fuel costs associated with replacing them with new fuels [22]. However, they ignore the health costs associated with the use of solid fuels. Here, we investigate whether the use of solid fuels causes a heavier burden on medical costs, and show whether it will be more expensive in terms of total expenditure.

Second, we discuss why the burden of health care expenditures is higher for households that use solid fuels. We tested two possible mechanisms. The first mechanism tests whether the use of solid fuels reduces the health level of household members. Regarding physical health, we first use the health level, as judged by the interviewer, to represent their health status. In the micro survey data, respondents’ health status is scored by the interviewers, with a score of 1 indicating the least healthy and 7 indicating the healthiest. Since tens of thousands of households receive judgments from different interviewers, the criteria for assessing health status vary among interviewers. If the health status is incorrect due to subjective judgment, the health impact is then considered in aggregate by considering whether the household member has recently been affected by discomfort, chronic illness, bronchitis or hospitalization due to illness. On the other hand, households using clean fuels may pay higher fuel costs, which has a crowding-out effect on health care expenditures when disposable income is certain. Thus, it shows that households with clean fuels have a lower share of health care expenditures. Therefore, we tested whether there is a significant difference in fuel costs between households using stationary and non-solid fuels.

Third, we study the heterogeneity between different types of households. Solid fuel use is more common in rural than in urban areas. Solid fuel use in rural areas may cause spillover, and households living in rural areas are affected not only by their own fuel but also by their neighbors’ fuel. Whether or not households own their own house can affect their willingness to change cooking fuels. When people temporarily live in someone else’s house, they are reluctant to invest too much in the interior amenities of the house and may be less likely to adopt indoor air pollution avoidance measures. On average, residents with different levels of education have different perceptions of solid fuels and differ in their living habits and attitudes toward medical care. The impact of fuel on health care expenditures may differ between groups, so we considered heterogeneity across groups. To enhance the robustness of our conclusions, we use the Ordinary Least Squares method, Propensity Score Matching method, Tobit model, Two-stage Least Squares method and other empirical methods to avoid some of the potential problems.

The remainder of this paper is organized as follows. Section 2 briefly reviews the previous related studies. The survey data and empirical methods are presented in Section 3. In Section 4, we discuss the results. Section 5 provides the mechanism analysis and heterogeneity analysis. Section 6 concludes with the main findings.

## 2. Literature Review

### 2.1. Household Fuel Use

Households in China all have current access to electricity. Still, due to the wealth inequality, economic development, geographical location, resource endowments and other reasons, the energy consumption of households across China is quite different. If we only focus on energy consumption or other economic level indicators, we may ignore the individual differences [23]. Based on individual circumstances, Belaïd proposed the 10% indicator, LIHC indicator and other indicators to calculate energy consumption [24]. The Stochastic Model of Energy Poverty (SMEP) was developed to calculate the required energy cost in a family [25]. With the SMEP method, Papada and Kaliampakos developed a new index, DCEN (Degree of Coverage of Energy Needs), which was expressed as the ratio of actual energy cost and required energy cost [26]. The aspect of compression of energy needs showed a household’s inability to meet its energy needs. Another way to determine the energy poverty is the comparison with surroundings. Karpinska and Śmiech obtained an estimation of the expected consumption of energy in households [27]. They then compared the disposable income after deducting the expected energy costs with a national median. Teschnera et al. compared two EU countries in terms of national norms, policies and regulation with references to energy poverty [28]. They concluded by suggesting that policymakers adopt more measures to promote clean energy.

Deprivations on availability and affordability of clean energy often happen simultaneously, thus studies have assessed the extent of multidimensional energy poverty [29]. Some studies used two steps to identify multidimensional poverty. The first step was to examine whether a person was deprived within each dimension, and the second step was to count the dimensions in which a person was deprived [30]. Other studies measured the degree, determinants and trend of Ghana’s multidimensional energy poverty and discussed the relationship between energy poverty and income [29,31].

Currently, an energy conversion in China’s residential sector is underway. Lin and Wang measured China’s energy poverty from the perspective of affordability. According to the Chinese General Social Survey (CGSS), several measurement indexes of energy poverty, including LIHC, 10% indicator and minimum energy demand, demonstrated that the degree of energy poverty in the central and western regions are greater [15]. Zhang et al. used the dual perspectives of accessibility and affordability to construct comprehensive indicators that accounted for access to clean fuel for cooking and economic constraints on using different forms of energy to illustrate the current status of multidimensional energy poverty in China [17].

We can see a significant reduction of energy poverty, but the incidence remains high. Therefore, we need to better understand the determinants of energy poverty [32]. Studies have shown that off-farm employees preferred using cleaner energy instead of traditional solid energy. Skills or literacy training were helpful in promoting the use of clean energy. The ecological values had positive effects on energy-saving behaviors, but did not have any effect on the choice of clean fuel [33]. For tackling energy poverty, we should take into account six challenges: quality of the dwelling fabric, energy costs and supply issues, stability of household income, tenancy relations, social relations within the household and outside, and ill health [34]. In terms of convenience and cleanliness, the boiling time of alternative fuels and the concentration of polluting particles in the room can affect the replacement of stove cookstoves [35]. Family size, income, education backgrounds, coal price and female labor participation are all influential in households’ fuel choice [36,37,38]. Higher levels of education, younger and higher income households will use LPG over kerosene; misperceptions about benefits, safety, and costs are important barriers to LPG use. Concerns on safety and high costs are potential barriers [22]. After going through the fuel replacement program, 34% of households still do not use clean cookstoves, and reasons associated with this include women’s education, age, and status, household wealth and family size, region, ability to use clean cookstoves, availability of clean cookstoves and subsidy levels. When pursuing policies, attention should be paid to household characteristics, household replacement needs and the knowledge level of the communities [39]. We should adopt strategies to facilitate the access to clean fuel and reduce multidimensional energy poverty. Recommendations to raise awareness and incentives for using clean fuels are factors influencing the choice of household fuels [40]. Educational interventions may be a cost-effective approach that should be carried out through community-based interventions [22].

### 2.2. Impact of Solid Fuels

To identify the correlation between air pollution and health, some scholars use randomized controlled trials. Mortimer et al. found that replacing solid fires with cleaner cookstoves would reduce the pneumonia incidence of children in rural Malawi, but the effect is not significant [41]. While another series of trials held in Guatemala showed that lower indoor wood smoke emissions were associated with reductions of certain severe pneumonia [42]. Transition from solid fuel to cleaner fuel reduced negative pregnancy outcomes because household air pollution exposure could significantly affect birthweight, preterm delivery and miscarriage [43]. The randomized controlled trials have a high level of internal validity.

Some other studies use survey data and adopt instrumental variable methods to overcome endogeneity. Silwal and McKay used the distance to nearest markets as an instrumental variable of solid fuel use, and found switching to cleaner cooking fuel improved lung capacity. The effect was larger among women and children [44]. Yang et al. conducted a population-based cohort study, and obtained pollutant data from the air exposure monitors nearest to pregnant women’s residence. The results revealed that exposure to high levels of pollutants increase the risk of stillbirth [38]. Household smoke-exposure risks can be defined by cooking fuels and cooking places; 76.4% of households Tanzania had high smoke-exposure risk when using smoky fuels for indoor cooking [45].

Studies on the health effects of only several pollutant measurements may raise the omitted variable bias, because health problems can be driven by other invisible pollutants. An exogenously given policy can provide a solution. Imeda confirmed that under the fuel-switching program implemented in Indonesia, households switching to clean fuels could effectively reduce premature deaths and underweight conditions. Since there was no way to measure the degree of indoor air pollution directly, the author used other research results to compare the indoor air quality when different fuels were used [46]. Liu et al. focused on the impact of household cooking fuel on the daily activities of the elderly. They used propensity score matching and endogenous conversion regression models to avoid sample selection bias. They found that the elderly in households that used non-solid fuels could better deal with daily life, and women were affected more [18]. Women who cooked with clean fuels were significantly less likely to suffer from chronic or acute diseases compared to those who cooked with solid fuels [47]. Children are more susceptible to indoor air and the use of solid fuels in the home can cause acute respiratory problems in children [48].

The impacts of energy poverty on a household’s well-being are enormous [49]. Studies examining clean fuel use have mostly focused on environmental and health studies, but few have evaluated the time saved. One such study revealed the time savings (1.5–1.9 h per day) in collecting fuel and cooking could be translated to an increase in income through work, showing a 3.8–4.7% increase in daily income [50]. The probability of coughing, shortness of breath, fever, acute respiratory infections and severe respiratory infections was higher when the cooking fuel was wood and lower when charcoal was used. Families using solid fuels have a higher probability of observing angina pectoris [51]. The variation in systolic blood pressure was higher with solid cooking fuels. This difference is not influenced by dietary intake [52]. Energy poverty negatively impacted households’ average school years and health status and the lack of access to clean energy posed a more severe challenge to health [6,17]. Effective policy measures and low-carbon sustainable technologies are required to create better living conditions [53,54].

### 2.3. Literature Summary

There have been many studies on the relationship between household fuels and health. Household fuel replacement is influenced by a variety of factors. Reasons external to the household include the strength of subsidies, the dissemination of knowledge about it and the safety and cost of the fuel itself. Intra-household reasons include household income, the jobs of household members, age and education level. Households that use solid fuels spend more time collecting fuel. More importantly, the use of solid fuels can increase indoor air pollution. Therefore, some existing research discuss the effects of using solid fuels on physical health, acting ability and mental health. However, in the micro survey data, the physical and mental health level is usually assessed by respondents or interviewers. The criteria of scores varies from person to person, thus in the micro survey data, physical and mental health is a subjective measure. In contrast, medical expenses can directly reflect the actual consumption decisions of residents about how much people pay for health care. There are some studies that investigate the effects of environmental issues, health insurance and treatment-seeking behavior on health care spending, but seldom investigate the impact of solid fuel use. Therefore, this article focuses on medical expenditures and tests whether solid fuel use adds to the burden of health expenses on households. In addition, high medical expenses are presently plaguing people, and many families are impoverished by illness. This article further explores whether the implementation of energy poverty alleviation measures can reduce the medical expense burden for families.

## 3. Materials and Methods

### 3.1. Data Sources

This article uses data from China Family Panel Studies (CFPS). The CFPS traces data from three levels: communities, families and individuals. It portrays the development of Chinese society, population, economy, education and health. Additionally, it provides reliable micro-data support for related academic research and policy analysis. The Institute of Social Science Survey of Peking University implements the CFPS. Its pre-surveys were conducted in 2008 and 2009. The formal baseline survey started in 2010, and a full sample survey was conducted every two years. The latest released data is for 2018. The CFPS formal survey samples cover 25 provinces, cities and autonomous regions (except Inner Mongolia, Hainan, Tibet, Qinghai, Ningxia, Xinjiang, Hong Kong, Macao and Taiwan), representing 95% of China’s population. The database structure includes four main questionnaire types: community questionnaire, family questionnaire, adult questionnaire and child questionnaire.

This paper merges family and individual databases, and obtains both family characteristics and individual characteristics. Firstly, we select fuel usage, medical expense, total consumption expense and other family characteristics for needs in the household database. Then, we use the individual database to match the member information corresponding to each family. Table 1 shows the definition of selected variables. Deleting cases with missing variables, our collated data include 9960 households and 28,976 individual observations. The main explanatory variable used in this paper denotes the main household cooking fuels to distinguish whether households use solid fuels. In the survey sample, the highest percentages of solid fuel use are in the provinces of Gansu, Jilin, Anhui, Shaanxi and Sichuan. The provinces with the lowest percentages of solid fuel use are Tianjin, Beijing, Shanghai, Zhejiang and Jiangsu. The burden of medical expenditures is the dependent variable, expressed as the proportion of medical expenses in total household expenditure. The average medical burden is 11.452%, while the average proportion of fuel fee is 0.247%. Fuel expenditures represent a small share of household consumption expenditures compared to medical expenditures. A total of 72.1% of households use tap water for cooking, and 70.9% (=1 − 0.291) of households use non-solid fuels. In this sample, 48.2% of households live in urban areas. We then compared the difference between urban and countryside areas.

Both assets and income play an important role in household consumption decisions. In consumption theory, there is a positive relationship between consumption and disposable income. However, in China, whether people consume or save depends to a large extent on the family’s assets. When assets are low, households increase precautionary savings and reduce consumption expenditures. Therefore, we controlled the asset and income variables separately during our empirical tests. This study includes other variables affecting household fuel choice, including education years, family size and age. Moreover, several variables have been chosen to represent the health status of residents.

### 3.2. Model Design

Based on the review of existing literature, we firstly identified the relationship between solid fuel use and the burden of household medical expenses. This paper sets the following baseline estimation model:

(1) $$Burden_{i}=\alpha +\beta \cdot Fuel\_solid_{i}+\gamma X_{i}+\lambda_{j}+\mu_{i}$$

where $Burden_{i}$ indicates family medical expense burden.$\;Fuel\_solid_{i}$ is a dummy variable of solid fuel, and its value is 1 when the household mainly uses solid fuel for cooking, otherwise the value is 0. $X_{i}$ means the control variables of the family. $\lambda_{j}$ is the province fixed effect. $\mu_{i}$ is an unobservable error.

There is a potential concern of self-selection bias. It is possible that solid fuels are more likely to be used by economically disadvantaged households, and these families have a higher burden of medical expenditures because of their lower income. There may be significant differences in some economic variables between households using solid fuels and non-solid fuels, resulting in households not being randomly assigned between the two groups. Perhaps the self-selection bias would make the estimated results biased. Therefore, referring to the methods of former research [55,56], we used the Propensity Score Matching (PSM) method for validation. The core idea of PSM is to use statistical techniques to artificially construct a control group by trying to match each participant (treated) with an untreated group by those observable characteristics. In other words, for observable variables, the control group constructed by matching has the same random distribution as the treatment group. 

We used covariates to estimate the propensity score of using solid fuels, and established a control group with similar observation characteristics as the treatment group. Comparing the statistical comparison group, we obtained the Average Treatment Effect on the treated group (ATT), namely

(2) $$ATT=E\left[Y_{i1}|p\left(X_{i}\right),\;Fuel\_solid_{i}=1\right]\;-\;E[Y_{i0}|p\left(X_{i}\right),Fuel\_solid_{i}=1]$$

where $p\left(X_{i}\right)$ is probability of choosing solid fuel, using observed characteristics $X_{i}$. $Y_{i1}$ is the outcome if individual *i* uses solid fuels, while $Y_{i0}$ is the outcome if individual *i* uses non-solid fuels. We can only observe one outcome in $Y_{i1}$ and $Y_{i0}$, because individual *i* is just in one status (using solid fuel, or not). Then we constructed the counterfactual outcome by PSM, and estimated the treatment effect.

In terms of the family medical expense burden, the sample data only consists of values equal to zero or bigger than zero. There is no value smaller than zero. Thus, family medical expense data is censored data. Zero is a censored point, and we can see many families have zero medical expense. The medical expense does not satisfy a normal distribution, so using the Tobit method will get a more accurate estimation.

In the censored data, we set up the latent variable of family medical expense. The latent variable satisfies the normal distribution, but is unobservable. When the latent variable is smaller than zero, the observed value of medical expense is zero. Therefore, based on the baseline model, consider setting up the following Tobit model:

(3) $$Burden_{i}^{*}=\alpha +\beta \cdot Fuel\_solid_{i}+\gamma X_{i}+\lambda_{j}+\mu_{i}\;Burden_{i}=Burden_{i}^{*},\;if\;Burden_{i}^{*}>0\;Burden_{i}=0,\;if\;Burden_{i}^{*}<0$$

where $Burden_{i}$ indicates family medical expense burden, $Burden_{i}^{*}$ is the unobservable variable or latent variable. Other variables have the same meanings as the baseline model (1). The most concerning factor in this model is the coefficient β, reflecting whether medical expense is affected by solid fuels.

This baseline model may have endogeneity bias due to missing variables. To deal with this problem, we used a variable indicating whether tap water was being used for cooking as an instrumental variable. The instrumental variable *water_tap* is a dummy variable. Its value is 1 when the household uses tap water for cooking, and its value is 0 when the household uses well water, lake water, spring water or rainwater. Households that have access to tap water have better access to modern clean cooking fuels. Because access to tap water reflects the local infrastructure, which is not affected by the characteristics of the household. Thus, using tap water as an instrumental variable satisfies both the conditions of relevance and exogeneity. The Two-Stage Least Squares Model replaces the endogenous variable with the predicted value, which is generated by instrumental variables. Based on the baseline model and the Tobit model, we implemented a Two-stage Least Squares Model (2SLS):

(4) $$Fuel\_solid_{i}=\alpha^{\prime }+\beta^{\prime }\cdot Water\_tap_{i}+\gamma^{\prime }X_{i}+\lambda_{j}+e_{i}\;Burden_{i}^{*}=\alpha +\beta \cdot \hat{Fuel\_solid_{i}}+\gamma X_{i}+\lambda_{j}+\mu_{i}$$

In the first stage, a regression is performed on the equation containing only the exogenous variables. Second, regression prediction of the endogenous variable $\hat{Fuel\_solid_{i}}$ is generated, and then the predicted value is used in place of the endogenous variable. 

## 4. Empirical Results and Discussion

### 4.1. Analysis of Baseline Regression

In this section, based on the empirical data and the multiple methods mentioned above, we examine the relationship between household medical expenditures and solid fuel use, and consider the effects of household characteristic variables. Table 2 shows empirical results of baseline regression. We include region fixed effect in each column, to control factors that are not observed and do not change over time, such as cultural characteristics unique to a region. In column (1) and (2), we controlled fixed effect at the province level. In column (3) and (4), province and county fixed effects are both controlled. We further add community fixed effect in column (5) and (6). Considering the different impacts of income and asset, we used them separately in the empirical process.

In the baseline OLS regression model, after controlling households’ characteristic variables, the coefficient of *Fuel_solid* in Table 2 is significantly positive, which means that households using solid fuels have a heavier medical expense burden. Since the burden of medical expense is measured by the proportion of medical expense in the total household expenses, the coefficient of *Fuel_solid* indicates that when solid fuel is used, the proportion of medical expense in all expenses will increase by 1.4–1.9%. From the above descriptive statistics of the data, we found that the proportion of medical expense in all expenses is about 10% on average, so 1.4–1.9% is a big change. For example, in column (6), compared to the average medical burden 10.191% in households using non-solid fuels, using solid fuels would increase the proportion to 11.954% (=10.191% + 1.763%). The change rate is 17.3% (=1.763/10.191 × 100%). Therefore, the economic costs due to the use of solid fuels cannot be ignored. Promoting the replacement of solid fuels with clean fuels in residential households can significantly reduce the expenditure on health care.

With a significant negative relationship between years of education and health care expenditure burden, we can speculate that people with higher levels of education may have more access to information about the effects of cooking fuels and will act to tackle circumvent air pollution [22,40]. The share of medical expenditures increases when the average age of household members is older. As people age, they are more likely to spend more on health care as their physical capabilities decline [39]. Household size has an inverse relationship with household health care expenditures. This is because when household wealth is constant, larger households have more pressure on consumption spending and are likely to pay less attention to health aspects and try to avoid medical care if they have only minor illnesses [39]. The coefficient of *exercise* is significantly negative at 1% level, while the coefficient of *exercise time* is insignificant. The more often people exercise during a week, the fewer medical expenses people will pay; it is the act of exercising, not the length of time spent on exercising, that plays a major role. Medical expenditures increase when the level of health decreases, when there are uncomfortable symptoms and when there are chronic diseases. Among the control variables indicating health, the coefficient of *hospitalized* is the largest. This is because in China, hospitalization costs account for the bulk of household medical expenditures. Both assets and income, which indicate the level of wealth, are negatively correlated with health care spending. This is because an increase in wealth level can increase total household consumption expenditure [40]. 

### 4.2. Estimation by Propensity Score Matching

There may be a problem of self-selection in the use of solid fuels, since people with relatively poorer conditions are more inclined to solid fuel. The method of Propensity Score Matching (PSM) is adopted here to reduce the bias caused by self-selection. At first, we chose the covariates from observable characteristic variables above, and conducted a balance test. The standardized bias is an indicator of the data balance of two groups, to show whether there is no significant difference between the treatment group and constructed control group. After matching, the absolute values of standard biases are reduced to less than 10%, which show the two groups do not have a standardized difference for each variable. Moreover, the *t*-test and *p*-value support the quality of matching. 

Table 3 shows a variety of matching methods used to compute the propensity score, for which robust results have been obtained. Judging from the estimated results of the one-to-one matching, the estimated results of ATT, ATU and ATE are all significantly positive, indicating that the medical expense burden of households using solid fuels is heavier. Similar conclusions are obtained by a variety of matching methods, indicating the estimation results’ robustness. The coefficients after matching are in the range of 1.5% to 2.2%, which is slightly smaller than the unmatched effect 4.6%. The results are consistent with the empirical estimates above. After matching by observable characteristics, the 1.5–2.2% increase related to solid cooking use deserve attention, since the average proportion of medical burden is about 10%.

### 4.3. Results Discussion of Tobit and 2SLS Methods

As we show in the research design above, the observations of medical expense are censored because there are many 0 values. Tobit method is used here for more accurate estimation. In Method (i) of Table 4, it can be observed that after considering more robust methods, households using solid fuels still have a heavier burden on family medical expenses, and the estimated coefficients are 1.4–1.9%, which are consistent with the above results.

Even if the families’ characteristics are controlled, there may still be missing variables, so we use *water_tap* as the instrumental variable here. Households that use tap water for cooking have better access to modern clean cooking fuels, because access to tap water reflects the local infrastructure. However, water infrastructure is not affected by the characteristics of the household. Thus, using tap water as an instrumental variable satisfies both the conditions of relevance and exogeneity. The coefficients in Method (ii) and (iii) are larger than that of the OLS method, but this is a common situation in the 2SLS method. Using tap water as an instrumental variable for using solid cooking fuels, the relationship between solid fuel use and medical expense is still significantly positive.

## 5. Mechanism and Heterogeneity Analysis

### 5.1. Mechanism Analysis

Studies have shown that solid fuel can cause indoor air pollution and harm residents’ health. According to existing studies about how solid fuels affect the burden of household medical expenses, households using solid fuels will suffer greater indoor air pollution. Meanwhile, collecting solid fuels and using solid fuels require more housework and labor time, so people rarely have time to relax and exercise [6,18,46,50]. 

Through the mediation effect test, this paper verifies the influence mechanism. In Table 5, the coefficients of *fuel_solid* on *health* are significantly negative. It demonstrates that the use of solid fuels will reduce family members’ health, thereby increasing the medical expenses burden. Households have high smoke-exposure risk when using smoky fuels for indoor cooking [45]. 

In (ii), (iii) and (iv), the coefficients of *fuel_solid* are significantly positive. When households use solid cooking fuels, the probability of health problems increase. The variation in systolic blood pressure was higher with solid cooking fuels [52]. Women who cooked with clean fuels were significantly less likely to suffer from chronic diseases compared to those who cooked with solid fuels [47]. Using solid cooking fuels in the home can cause bronchitis, including acute respiratory problems in children [48]. Families using solid fuels have a higher probability of observing angina pectoris [51]. Overall, the use of solid fuels places a heavy health burden on households [57].

In the process of promoting clean fuel conversion, some households are unwilling to use clean fuels. Even if they have access to clean fuels, such as electricity and natural gas, they usually use solid fuels for cooking because some may think that modern energy costs more, and they do not recognize the harm caused by solid fuels. Due to the limited disposable income and budget constraints, the proportion of fuel costs may squeeze out the proportion of medical expenses. The lower medical burdens for families who use the modern non-solid fuels may be due to higher proportions of fuel costs when the total expenditures remain unchanged. Therefore, it was tested whether fuel switching affects the proportion of fuel fee in total expenses. There was found to be no significant impact in Table 6, which is similar to the result of Imeda [46] because the fuel expense itself accounts for a relatively small proportion of the total expense. In Table 1, the proportion of fuel fee in total expense was just 0.247%, much lower than the proportion of medical expenses at 11.452%. Even if the fuel is replaced, the proportion of fuel expense does not change significantly, and there is insufficient evidence to show that fuel expenses squeeze out medical expenses.

### 5.2. Heterogeneity Analysis

There are substantial subgroup differences in effects of energy use. Some studies revealed that the reduction of solid fuel use is related to household characteristics and the local policy changes [29]. The structure of a household is also influential. Single-person or one-parent households are mostly threatened by energy poverty. In addition, households exposed to energy poverty are more likely to live in remote rural areas or less urbanized regions [6,27,36]. In this paper, we divided the whole sample into subgroups according to household characteristics. Then, we used dummy variables (*urban*, *house* and *education*) to indicate which subgroup a family belonged to, and constructed the interaction term of the dummy variable and *fuel_solid*. A significant positive or negative coefficient of the interaction term indicates the impact of solid fuels on medical expenditures is moderated by the dummy variable. Table 7 shows the IV-Tobit estimates by including the intercept term.

Households in cities and towns may be less affected by solid fuels from surrounding households, which will weaken the impact of solid fuels on household medical expenses. Based on the above empirical model, the interaction terms of *urban* and *fuel_solid* were included to analyze the heterogeneity of urban and rural areas. In (i) of Table 7, the interaction terms are all significantly negative, indicating that compared with rural areas, solid fuels have a smaller impact on the medical burden in city areas. The promotion of urbanization will help reduce the impact of solid fuels on household medical expenses.

Family members who have full ownership of the house they live in will be more inclined to improve the interior of the house. People are reluctant to invest too much money and material resources in a house that they live in temporarily. Households with equity and households without equity may be affected differently by solid fuels. In (ii) of Table 7, we see that the interaction terms of *house* and *fuel_solid* are significantly negative, suggesting that households with ownership have more means to mitigate the impact of cooking fuels when faced with it.

The sample is divided into two groups according to educational levels. More educated people are able to search for more information, have a clearer understanding of cooking fuel use, and learn more ways to mitigate the effects of fuel. An interaction term of higher education *high* and solid fuel use *fuel_solid* was added to the above empirical model to analyze the heterogeneity of education. In (iii) of Table 7, we can see that the interaction terms are significantly negative, indicating that increasing the education level of residents can also help reduce the burden of medical expenses caused by solid fuel use.

## 6. Conclusions

This article uses micro-survey data to examine the relationship between solid fuel use and the burden of medical expenses. The results show that the use of solid cooking fuels will increase the burden of family medical expenses. We used the Propensity Score Matching method, Tobit method, and Instrumental Variable method, and the results obtained are robust. Through the examination of the impact mechanism, we found that the negative effect of solid cooking fuels on medical expense is mainly due to the poorer health status. However, fuel transition does not significantly affect the proportion of fuel costs in the total household expenditure. Compared with less educated rural households without house equity, better educated urban households with house equity suffer less from medical expenses caused by solid cooking fuels. It shows that the effect of solid cooking fuel on medical expenses is greater in the disadvantaged groups.

In recent years, the proportion of solid fuel use in households has been steadily declining. Governments should further promote the conversion from solid fuels to clean and modern fuels, which will help to improve residents’ health and reduce their’ medical expense burden. The fuel transition in the residential sector also contributes to low-carbon use and the sustainable development of society. We need to continue to promote urbanization and improve the education level of residents. Urbanization not only promotes the use of non-solid fuels, it also brings more comprehensive and convenient public facilities that are beneficial to the health of residents. Improving residents’ education level can improve their healthy awareness and, to a certain extent, reduce the burden of medical expenses. It is necessary to propagate the benefits of fuel switching and encourage residents to replace solid fuels. The changes in fuel expenses due to fuel conversions do not account for a large proportion of the total household expenses. However, they have a greater impact on medical expenses. Therefore, the use of clean fuels can benefit health and save money overall. The government could provide appropriate subsidies to allow more residents to use clean fuels and improve residents’ welfare.

There are some limitations of this paper that could be addressed in future research. Based on the household survey data, we can only know what the main cooking fuel is, but not how much is being used; thus, we could not accurately measure the indoor air pollution in households. In addition, although we have considered the health status and illness of family members, we could not distinguish different types of medical expenditures. Data with more detailed information could be used in the future. Furthermore, we conclude that the benefits of promoting clean fuels outweigh the disadvantages. However, in the process of promoting clean fuels, some residents are still unwilling to replace solid fuel with non-solid fuel; therefore, further research is needed on how to promote fuel conversion more effectively.

## Figures and Tables

**Table 1:** Descriptive statistics of household fuel use and related characteristics.

Variables	Explanation	(1)	(2)	(3)
		N	Mean	SD
burden	Proportion of medical cost in all expense (%)	9960	11.452	20.857
fuelfee_ratio	Proportion of fuel fee in all expense (%)	9960	0.247	0.536
fuel_solid	Solid fuel as main cooking fuel (=1); Otherwise (=0)	9960	0.291	0.454
water_tap	Tap water as main cooking water (=1); Otherwise (=0)	9960	0.721	0.449
urban	Living in an urban area (=1); Otherwise (=0)	9960	0.482	0.500
ln(asset)	Logarithm of net assets (RMB)	9960	12.576	1.409
ln(income)	Logarithm of income (RMB)	9960	9.108	1.739
Familysize	Family size	9960	3.790	1.896
eduy	Average years of education of family members	9960	7.332	3.866
age	Average ages of family members	9960	48.517	14.155
exercise	Average frequency of exercise in a week	9960	2.819	2.682
exercisetime	Average time of exercise in a week	9960	4.521	6.956
health	Average health level of family members judged by interviewer (1, lowest; 7, highest)	9960	5.443	1.216
uncomfortable	Has been unwell in the past two weeks (=1); Otherwise (=0)	9960	0.549	0.498
chronic	Any chronic diseases within six months (=1); Otherwise (=0)	9960	0.347	0.476
bronchitis	Any bronchitis within six months (=1); Otherwise (=0)	9960	0.115	0.319
asthma	Any asthma within six months (=1); Otherwise (=0)	9960	0.055	0.228
hospitalized	Any hospitalization due to illness in the last 12 months (=1); Otherwise (=0)	9960	0.290	0.454

Note: The above measures are based on the CFPS data.

**Table 2:** Baseline regression results of solid cooking fuels on medical expense burden.

	(1)	(2)	(3)	(4)	(5)	(6)
fuel_solid	1.596 ***	1.913 ***	1.373 **	1.630 ***	1.600 **	1.763 **
	(0.509)	(0.498)	(0.644)	(0.629)	(0.775)	(0.768)
eduy	−0.208 ***	−0.229 ***	−0.174 ***	−0.183 ***	−0.132	−0.141 *
	(0.059)	(0.061)	(0.064)	(0.066)	(0.082)	(0.085)
age	0.184 ***	0.187 ***	0.179 ***	0.182 ***	0.186 ***	0.189 ***
	(0.018)	(0.018)	(0.018)	(0.018)	(0.023)	(0.023)
exercise	−0.246 **	−0.255 ***	−0.222 **	−0.228 **	−0.331 ***	−0.334 ***
	(0.098)	(0.095)	(0.096)	(0.094)	(0.123)	(0.122)
exercisetime	0.013	0.010	0.006	0.003	0.014	0.011
	(0.035)	(0.034)	(0.034)	(0.033)	(0.038)	(0.038)
health	−1.731 ***	−1.782 ***	−2.240 ***	−2.301 ***	−2.779 ***	−2.848 ***
	(0.269)	(0.257)	(0.341)	(0.324)	(0.391)	(0.378)
uncomfortable	1.611 ***	1.666 ***	1.572 ***	1.616 ***	1.441 ***	1.472 ***
	(0.385)	(0.387)	(0.388)	(0.388)	(0.444)	(0.445)
chronic	1.833 ***	1.840 ***	1.732 ***	1.741 ***	2.140 ***	2.139 ***
	(0.535)	(0.531)	(0.550)	(0.547)	(0.560)	(0.560)
bronchitis	0.569	0.624	0.732	0.777	0.809	0.843
	(0.937)	(0.939)	(0.947)	(0.947)	(0.986)	(0.987)
asthma	−0.086	−0.163	−0.185	−0.269	−0.491	−0.581
	(1.160)	(1.164)	(1.159)	(1.159)	(1.279)	(1.277)
hospitalized	9.124 ***	9.095 ***	8.963 ***	8.932 ***	9.057 ***	9.032 ***
	(0.581)	(0.582)	(0.593)	(0.594)	(0.660)	(0.661)
familysize	−0.278 **	−0.379 ***	−0.301 **	−0.391 ***	−0.290 **	−0.386 ***
	(0.120)	(0.123)	(0.125)	(0.129)	(0.144)	(0.149)
ln(asset)	−0.791 ***		−0.723 ***		−0.805 ***	
	(0.223)		(0.246)		(0.287)	
ln(income)		−0.474 ***		−0.455 ***		−0.360 **
		(0.129)		(0.133)		(0.158)
Province fixed	Y	Y	Y	Y	Y	Y
County fixed			Y	Y	Y	Y
Community fixed					Y	Y
N	9960	9960	9958	9958	9730	9730
r2	0.133	0.132	0.154	0.154	0.232	0.232
r2_a	0.129	0.129	0.131	0.131	0.103	0.103
Control Mean	10.120	10.120	10.120	10.120	10.191	10.191

Note: Standard errors in parentheses. *** *p* < 0.01, ** *p* < 0.05, * *p* < 0.1.

**Table 3:** Results of PSM estimation on treatment effect of solid cooking fuel use.

	(1)	(2)	(3)	(4)	(5)	(6)	(7)
Matching Methods	One-to-One	Neighbor	Caliper	Radius	Kernel	Local Linear	Mahal
Un-matched	4.584 ***	4.584 ***	4.584 ***	4.584 ***	4.584 ***	4.584 ***	4.584 ***
	(0.458)	(0.458)	(0.458)	(0.458)	(0.458)	(0.458)	(0.458)
ATT	2.223 **	1.75 **	1.749 *	1.606 **	1.714 **	1.606 **	1.780 ***
	(0.944)	(0.842)	(0.912)	(0.684)	(0.759)	(0.703)	(0.658)
ATU	1.824 **	1.469 **	1.469 **	1.444 ***	1.689 ***	1.566 **	1.977 ***
	(0.743)	(0.653)	(0.679)	(0.522)	(0.597)	(0.618)	(0.625)
ATE	1.941 ***	1.552 **	1.552 **	1.492 ***	1.696 ***	1.578 ***	1.920 ***
	(0.602)	(0.605)	(0.626)	(0.494)	(0.603)	(0.578)	(0.580)
N	9960	9960	9960	9960	9960	9960	9960

Note: Standard errors in parentheses. *** *p* < 0.01, ** *p* < 0.05, * *p* < 0.1.

**Table 4:** Results of Tobit and 2SLS models.

	(1)	(2)	(3)	(4)	(5)	(6)
Method (i): Tobit estimates (medical expense as a censored variable)						
fuel_solid	1.596 ***	1.373 **	1.600 ***	1.913 ***	1.630 ***	1.763 ***
	(0.496)	(0.540)	(0.592)	(0.485)	(0.532)	(0.590)
Method (ii): 2SLS (tap water as an IV)						
fuel_solid	6.628 **	7.496 *	17.279 *	6.793 **	7.740 **	17.562 *
	(2.918)	(4.098)	(9.701)	(2.664)	(3.850)	(9.354)
Method (iii): IV-Tobit (tap water as an IV, and medical expense as a censored variable)						
fuel_solid	8.321 ***	9.606 **	20.714 **	8.250 ***	9.616 ***	20.715 **
	(2.989)	(3.913)	(8.562)	(2.730)	(3.679)	(8.280)
ln(asset)	Y	Y	Y			
ln(income)				Y	Y	Y
Other controls	Y	Y	Y	Y	Y	Y
Province fixed	Y	Y	Y	Y	Y	Y
County fixed		Y	Y		Y	Y
Community fixed			Y			Y
N	9960	9958	9370	9960	9958	9370

Note: Standard errors in parentheses. *** *p* < 0.01, ** *p* < 0.05, * *p* < 0.1.

**Table 5:** Effects of solid cooking fuels on health conditions.

	(1)	(2)	(3)	(4)	(5)	(6)
(i) health						
fuel_solid	−0.141 ***	−0.118 ***	−0.092 ***	−0.204 ***	−0.176 ***	−0.121 ***
	(0.034)	(0.032)	(0.031)	(0.033)	(0.032)	(0.031)
N	9960	9958	9370	9960	9958	9370
(ii) uncomfortable						
fuel_solid	0.051 ***	0.051 ***	0.050 ***	0.058 ***	0.056 ***	0.054 ***
	(0.012)	(0.014)	(0.015)	(0.012)	(0.013)	(0.015)
N	9959	9866	8892	9959	9866	8892
(iii) chronic						
fuel_solid	0.031 ***	0.027 **	0.032 **	0.033 ***	0.029 **	0.033 **
	(0.012)	(0.013)	(0.014)	(0.011)	(0.012)	(0.014)
N	9959	9854	8785	9959	9854	8785
(iv) bronchitis						
fuel_solid	0.023 ***	0.022 **	0.028 **	0.026 ***	0.024 ***	0.029 **
	(0.008)	(0.009)	(0.011)	(0.008)	(0.009)	(0.011)
N	9959	9763	7354	9959	9763	7354
ln(asset)	Y	Y	Y			
ln(income)				Y	Y	Y
Other controls	Y	Y	Y	Y	Y	Y
Province fixed	Y	Y	Y	Y	Y	Y
County fixed		Y	Y		Y	Y
Community fixed			Y			Y

Note: Standard errors in parentheses. *** *p* < 0.01, ** *p* < 0.05.

**Table 6:** Effects of solid cooking fuels on fuel fees.

	(1)	(2)	(3)	(4)	(5)	(6)
fuel_solid	−0.016	−0.009	−0.005	−0.007	−0.001	−0.000
	(0.013)	(0.020)	(0.014)	(0.014)	(0.022)	(0.014)
ln(asset)	−0.022 ***	−0.023 ***	−0.024 ***			
	(0.005)	(0.006)	(0.005)			
ln(income)				−0.016 ***	−0.016 ***	−0.014 ***
				(0.003)	(0.003)	(0.003)
Other controls	Y	Y	Y	Y	Y	Y
Province fixed	Y	Y	Y	Y	Y	Y
County fixed		Y	Y		Y	Y
Community fixed			Y			Y
N	9960	9958	9370	9960	9958	9370
r2	0.068	0.095	0.576	0.068	0.095	0.576
r2_a	0.065	0.071	0.505	0.065	0.071	0.505

Note: Standard errors in parentheses. *** *p* < 0.01.

**Table 7:** Heterogeneity analysis of effects of solid cooking fuels on medical expenses.

	(1)	(2)	(3)	(4)	(5)	(6)
(i) heterogeneity in urban (urban = 1, if living in urban areas; urban = 0, otherwise.)						
fuel_solid * urban	−9.022 *	−11.691 *	−35.671 **	−8.911 *	−11.731 *	−35.369 **
	(5.186)	−6.72	−17.383	−5.141	−6.693	−16.81
(ii) heterogeneity in house (house = 1, if house owned by household; house = 0, otherwise.)						
fuel_solid * house	−45.202 **	−45.092 **	−113.346 *	−45.077 **	−45.600 **	−113.769 *
	−21.373	(22.547)	(62.621)	−20.476	(22.006)	(61.329)
(iii) heterogeneity in education (education = 1, if higher than the average level; education = 0, if lower than the average level.)						
fuel_solid * education	−11.538 **	−11.075 **	−19.606 **	−11.480 **	−11.096 **	−19.599 **
	(4.577)	(4.554)	(8.124)	(4.469)	(4.477)	(7.983)
ln(asset)	Y	Y	Y			
ln(income)				Y	Y	Y
Other controls	Y	Y	Y	Y	Y	Y
Province fixed	Y	Y	Y	Y	Y	Y
County fixed		Y	Y		Y	Y
Community fixed			Y			Y
N	9960	9958	9370	9960	9958	9370

Note: Standard errors in parentheses. ** *p* < 0.05, * *p* < 0.1.

## Data Availability

Publicly available datasets were analyzed in this study. This data can be found here: [http://www.isss.pku.edu.cn/cfps/] (accessed on 16 April 2021).

## References

[1] IEA (International Energy Agency) (2020). World Energy Outlook 2020.

[2] Institute for Health Metrics and Evaluation (2016). Global Burden of Disease data tool.

[3] Du K., Shao S., Yan Z. (2021). Urban residential energy demand and rebound effect in China: A stochastic energy demand frontier approach. Energy J..

[4] Lin B., Wang Y. (2020). Analyzing the elasticity and subsidy to reform the residential electricity tariffs in China. Int. Rev. Econ. Financ..

[5] Niu S., Jia Y., Ye L., Dai R., Li N. (2016). Does electricity consumption improve residential living status in less developed regions?. An empirical analysis using the quantile regression approach. Energy.

[6] Oum S. (2019). Energy poverty in the Lao PDR and its impacts on education and health. Energy Policy.

[7] Dagoumas A., Kitsios F. (2014). Assessing the impact of the economic crisis on energy poverty in Greece. Sustain. Cities Soc..

[8] Quinn A.K., Bruce N., Puzzolo E., Dickinson K., Sturke R., Jack D.W., Mehta S., Shankar A., Sherr K., Rosenthal J.P. (2018). An analysis of efforts to scale up clean household energy for cooking around the world. Energy Sustain. Dev..

[9] IEA (International Energy Agency) (2002). Energy and Poverty: Special early excerpt of the World Energy Outlook 2002.

[10] Charlier D., Kahouli S. (2019). From residential energy demand to fuel poverty: Income-induced Non-linearities in the Reactions of Households to Energy Price Fluctuations. Energy J..

[11] Li K., Lloyd B., Liang X.J., Wei Y.M. (2014). Energy poor or fuel poor: What are the differences?. Energy Policy.

[12] Zhu J., Hu S., Wang J., Zheng X. (2020). Future orientation promotes climate concern and mitigation. J. Clean. Prod..

[13] Mastropietro P. (2019). Who should pay to support renewable electricity? Exploring regressive impacts, energy poverty and tariff equity. Energy Res. Soc. Sci..

[14] Zhu J., Wang J. (2021). The effects of fuel content regulation at ports on regional pollution and shipping industry. J. Environ. Econ. Manage..

[15] Lin B., Wang Y. (2020). Does energy poverty really exist in China? From the perspective of residential electricity consumption. Energy Policy.

[16] Tang X., Liao H. (2014). Energy poverty and solid fuels use in rural China: Analysis based on national population census. Energy Sustain. Dev..

[17] Zhang D., Li J., Han P. (2019). A multidimensional measure of energy poverty in China and its impacts on health: An empirical study based on the China family panel studies. Energy Policy.

[18] Liu Z., Li J., Rommel J., Feng S. (2020). Health impacts of cooking fuel choice in rural China. Energy Econ..

[19] Farrukh M., Khreis H. (2021). Monetizing the burden of childhood asthma due to traffic related air pollution in the contiguous united states in 2010. Int. J. Environ. Res. Public Health.

[20] Pi T., Wu H., Li X. (2019). Does air pollution affect health and medical insurance cost in the elderly: An empirical evidence from China. Sustain..

[21] Liu Z., Pagoulatos A., Hu W., Schieffer J. (2014). Valuing the Benefit of Reducing Adverse Effects from Polluting Heating Fuels. Soc. Sci. Q..

[22] Ozoh O.B., Okwor T.J., Adetona O., Akinkugbe A.O., Amadi C.E., Esezobor C., Adeyeye O.O., Ojo O., Nwude V.N., Mortimer K. (2018). Cooking fuels in lagos, Nigeria: Factors associated with household choice of kerosene or liquefied petroleum gas (LPG). Int. J. Environ. Res. Public Health.

[23] Day R., Walker G., Simcock N. (2016). Conceptualising energy use and energy poverty using a capabilities framework. Energy Policy.

[24] Belaïd F. (2018). Exposure and risk to fuel poverty in France: Examining the extent of the fuel precariousness and its salient determinants. Energy Policy.

[25] Papada L., Kaliampakos D. (2016). Measuring energy poverty in Greece. Energy Policy.

[26] Papada L., Kaliampakos D. (2020). Being forced to skimp on energy needs: A new look at energy poverty in Greece. Energy Res. Soc. Sci..

[27] Karpinska L., Śmiech S. (2020). Invisible energy poverty? Analysing housing costs in Central and Eastern Europe. Energy Res. Soc. Sci..

[28] Teschner N., Sinea A., Vornicu A., Abu-Hamed T., Negev M. (2020). Extreme energy poverty in the urban peripheries of Romania and Israel: Policy, planning and infrastructure. Energy Res. Soc. Sci..

[29] Crentsil A.O., Asuman D., Fenny A.P. (2019). Assessing the determinants and drivers of multidimensional energy poverty in Ghana. Energy Policy.

[30] Alkire S., Foster J. (2011). Counting and multidimensional poverty measurement. J. Public Econ..

[31] Adusah-Poku F., Takeuchi K. (2019). Energy poverty in Ghana: Any progress so far?. Renew. Sustain. Energy Rev..

[32] Scarpellini S., Rivera-Torres P., Suárez-Perales I., Aranda-Usón A. (2015). Analysis of energy poverty intensity from the perspective of the regional administration: Empirical evidence from households in southern Europe. Energy Policy.

[33] Huang F., Liu J., Wang Z., Shuai C., Li W. (2020). Of job, skills, and values: Exploring rural household energy use and solar photovoltaics in poverty alleviation areas in China. Energy Res. Soc. Sci..

[34] Middlemiss L., Gillard R. (2015). Fuel poverty from the bottom-up: Characterising household energy vulnerability through the lived experience of the fuel poor. Energy Res. Soc. Sci..

[35] Ojo K.D., Soneja S.I., Scrafford C.G., Khatry S.K., LeClerq S.C., Checkley W., Katz J., Breysse P.N., Tielsch J.M. (2015). Indoor particulate matter concentration, water boiling time, and fuel use of selected alternative cookstoves in a home-like setting in rural Nepal. Int. J. Environ. Res. Public Health.

[36] Hou B.D., Tang X., Ma C., Liu L., Wei Y.M., Liao H. (2017). Cooking fuel choice in rural China: Results from microdata. J. Clean. Prod..

[37] Wei T., Zhu Q., Glomsrød S. (2014). Energy spending and household characteristics of floating population: Evidence from Shanghai. Energy Sustain. Dev..

[38] Yang S., Tan Y., Mei H., Wang F., Li N., Zhao J., Zhang Y., Qian Z., Chang J.J., Syberg K.M. (2018). Ambient air pollution the risk of stillbirth: A prospective birth cohort study in Wuhan, China. Int. J. Hyg. Environ. Health.

[39] Wolf J., Mäusezahl D., Verastegui H., Hartinger S.M. (2017). Adoption of clean cookstoves after improved solid fuel stove programme exposure: A cross-sectional study in three peruvian andean regions. Int. J. Environ. Res. Public Health.

[40] Mperejekumana P., Li H., Wu R., Lu J., Tursunov O., Elshareef H., Gaballah M.S., Nepo N.J., Zhou Y., Dong R. (2021). Determinants of household energy choice for cooking in Northern Sudan: A multinomial logit estimation. Int. J. Environ. Res. Public Health.

[41] Mortimer K., Ndamala C.B., Naunje A.W., Malava J., Katundu C., Weston W., Havens D., Pope D., Bruce N.G., Nyirenda M. (2017). A cleaner burning biomass-fuelled cookstove intervention to prevent pneumonia in children under 5 years old in rural Malawi (the Cooking and Pneumonia Study): A cluster randomised controlled trial. Lancet.

[42] Smith K.R., McCracken J.P., Weber M.W., Hubbard A., Jenny A., Thompson L.M., Balmes J., Diaz A., Arana B., Bruce N. (2011). Effect of reduction in household air pollution on childhood pneumonia in Guatemala (RESPIRE): A randomised controlled trial. Lancet.

[43] Alexander D.A., Northcross A., Karrison T., Morhasson-Bello O., Wilson N., Atalabi O.M., Dutta A., Adu D., Ibigbami T., Olamijulo J. (2018). Pregnancy outcomes and ethanol cook stove intervention: A randomized-controlled trial in Ibadan, Nigeria. Environ. Int..

[44] Silwal A.R., McKay A. (2015). The Impact of Cooking with Firewood on Respiratory Health: Evidence from Indonesia. J. Dev. Stud..

[45] Ahamad M.G., Tanin F., Shrestha N. (2021). Household smoke-exposure risks associated with cooking fuels and cooking places in tanzania: A cross-sectional analysis of demographic and health survey data. Int. J. Environ. Res. Public Health.

[46] (2020). Imelda Cooking that kills: Cleaner energy access, indoor air pollution, and health. J. Dev. Econ..

[47] Nie P., Sousa-Poza A., Xue J. (2016). Fuel for life: Domestic cooking fuels and women’s health in rural China. Int. J. Environ. Res. Public Health.

[48] Kilabuko J.H., Nakai S. (2007). Effects of cooking fuels on acute respiratory infections in children in Tanzania. Int. J. Environ. Res. Public Health.

[49] Phoumin H., Kimura F. (2019). Cambodia’s energy poverty and its effects on social wellbeing: Empirical evidence and policy implications. Energy Policy.

[50] Simkovich S.M., Williams K.N., Pollard S., Dowdy D., Sinharoy S., Clasen T.F., Puzzolo E., Checkley W. (2019). A systematic review to evaluate the association between clean cooking technologies and time use in low- and middle-income countries. Int. J. Environ. Res. Public Health.

[51] Tiwari I., Herr R.M., Loerbroks A., Yamamoto S.S. (2020). Household air pollution and angina pectoris in low-and middle-income countries: Cross-sectional evidence from the world health survey 2002–2003. Int. J. Environ. Res. Public Health.

[52] Bellows A.L., Spiegelman D., Du S., Jaacks L.M. (2020). The association of cooking fuel use, dietary intake, and blood pressure among rural women in China. Int. J. Environ. Res. Public Health.

[53] Khanna R.A., Li Y., Mhaisalkar S., Kumar M., Liang L.J. (2019). Comprehensive energy poverty index: Measuring energy poverty and identifying micro-level solutions in South and Southeast Asia. Energy Policy.

[54] Tu Z., Hu T., Shen R. (2019). Evaluating public participation impact on environmental protection and ecological efficiency in China: Evidence from PITI disclosure. China Econ. Rev..

[55] Smith J.A., Todd P.E. (2005). Does matching overcome LaLonde’s critique of nonexperimental estimators?. J. Econom..

[56] Rahut D.B., Ali A., Mottaleb K.A. (2017). Understanding the determinants of alternate energy options for cooking in the Himalayas: Empirical evidence from the Himalayan region of Pakistan. J. Clean. Prod..

[57] Ho E.W., Strohmeier-Breuning S., Rossanese M., Charron D., Pennise D., Graham J.P. (2021). Diverse health, gender and economic impacts from domestic transport of water and solid fuel: A systematic review. Int. J. Environ. Res. Public Health.

